# Enhancing Communication and Consent Using 3‐D Printed Models With a Pediatric Dental Patient Presenting With Odontogenic Keratocysts Associated With Gorlin Syndrome: A Case Report

**DOI:** 10.1111/scd.70257

**Published:** 2026-09-16

**Authors:** Romain Jacq, Margaux Picherit, Anne‐Charlotte Bas

**Affiliations:** ^1^ Dental Department, UFR Santé University of Rouen Normandy Rouen Cedex France; ^2^ Dental Department Martainville O+ CHU De Rouen Rouen France

## Abstract

**Aims:**

This case report aimed to describe the use of a patient‐specific 3D printed model to support communication and informed consent in a pediatric patient diagnosed with Gorlin syndrome, a rare condition frequently associated with multiple odontogenic keratocysts.

**Methods and Results:**

A 9‐year‐old girl with Gorlin syndrome presented with swelling of the maxilla and mandible. Imaging revealed two large cystic lesions. Conventional explanations using radiographs and CBCT slices failed to ensure adequate parental understanding, partly due to language barriers. A digital workflow was developed using free segmentation software (3D Slicer) and a low‐cost fused deposition modelling printer to produce life‐size maxillary and mandibular models, with cysts highlighted in color. The models were presented during consultation, enabling the family to visualize the extent of the lesions and their proximity to critical structures. According to parental feedback, the model made the pathology and surgical rationale “much clearer” than prior explanations. Informed consent was successfully obtained, and the family adhered to follow‐up and referral recommendations.

**Conclusion:**

This case demonstrates that patient‐specific 3D printed models can enhance comprehension, engagement, and trust in the care of children with complex conditions. Such models represent a feasible, low‐cost adjunct to the informed consent process in dentistry.

## 1 Introduction

Nevoid basal cell carcinoma syndrome, also known as Gorlin syndrome, is a rare autosomal dominant condition. It's prevalence ranges from 1 in 57 000 to 1 in 256 000, and a sex ratio of 1:1. It is characterized by multiple basal cell carcinomas of the skin and multiple odontogenic keratocysts (OKCs) of the jaws, among other abnormalities [1].

Children with Gorlin syndrome often develop new or recurrent cysts, so early identification is crucial [2]. Management must balance effective treatment of each lesion with the need to minimize cumulative surgical morbidity in a growing child [3].

Jaw OKCs occur in the majority of patients with Gorlin syndrome (present in at least 75%) and often manifest in childhood or adolescence [1]. The presence of multiple jaw cysts at a young age should prompt evaluation for Gorlin syndrome, even if skin lesions or other stigmata are absent [4].

3D visualization techniques—ranging from virtual reconstructions to physical printed models—allow complex anatomies to be appreciated more intuitively than conventional two‐dimensional imaging. 3D printing has emerged as a valuable innovation in medicine, benefiting both surgical planning and patient education [5]. In maxillofacial treatment, 3D prints are mainly used for surgical planning and the education of medical students [6].

Growing evidence suggests that 3D models enhance patient and family understanding during the informed consent process by providing a tangible and intuitive representation of the pathology and planned intervention. This is especially pertinent in pediatric cases, where the consent discussion involves surrogate decision‐makers (parents or guardians) [7]. Prior studies showed that 3D‐printed models used in patient education lead to better comprehension, reduced anxiety, and greater satisfaction with care [8]. A recent randomized trial by Khan et al. found that adult surgery patients who were counseled with 3D‐printed models felt more engaged in shared decision‐making and experienced a greater decrease in preoperative anxiety compared to those receiving standard counseling [9]. Weng et al. reported that in pediatric otolaryngology cases, adding patient‐specific 3D prints to the consent discussion helped parents visualize their child's anatomy and significantly improved understanding while reducing parental anxiety [10]. These findings align with a 2024 systematic review of 3D modeling in general pediatric surgery, which concluded that 3D prints are a useful tool for improving patient and family education across various pediatric subspecialties [11].

We present a case report of a pediatric patient with Gorlin syndrome and multiple jaw cysts, in which a 3D‐printed model was used to facilitate communication. To our knowledge, this is the first reported case in pediatric dentistry where 3D printing was explicitly used to enhance patient/family understanding and informed consent. We describe the digital workflow for generating the model and discuss the impact of this tool on the family's decision‐making process. To focus on the pediatric consent process, the surgical details were not described in depth.

## 2 Innovation Report and Case Presentation

Patient: A 9‐year‐old female was referred to our service due to swelling in her cheek that had persisted for a few days, without pain or fever. The patient had been seen at our clinic for several years, although her follow‐up has been irregular. During the initial medical interview, no significant health issues were reported. The patient's main concern was a gradual swelling of the right midfacial region.

### 2.1 Extraoral and Intraoral Examination

Swelling was observed in the anterior right region of the maxilla, resulting in a visible and palpable bulge beneath the orbit. The overlying mucosa appeared normal, with no ulceration. The teeth in the affected area showed no mobility, but percussion elicited mild sensitivity. The occlusion remained unchanged. No vestibular fistula or fluctuation was present.

Oral hygiene was poor, with visible plaque accumulation and gingival inflammation. Multiple cavities were observed, affecting both primary and permanent teeth.

### 2.2 Imaging

**FIGURE 1:**
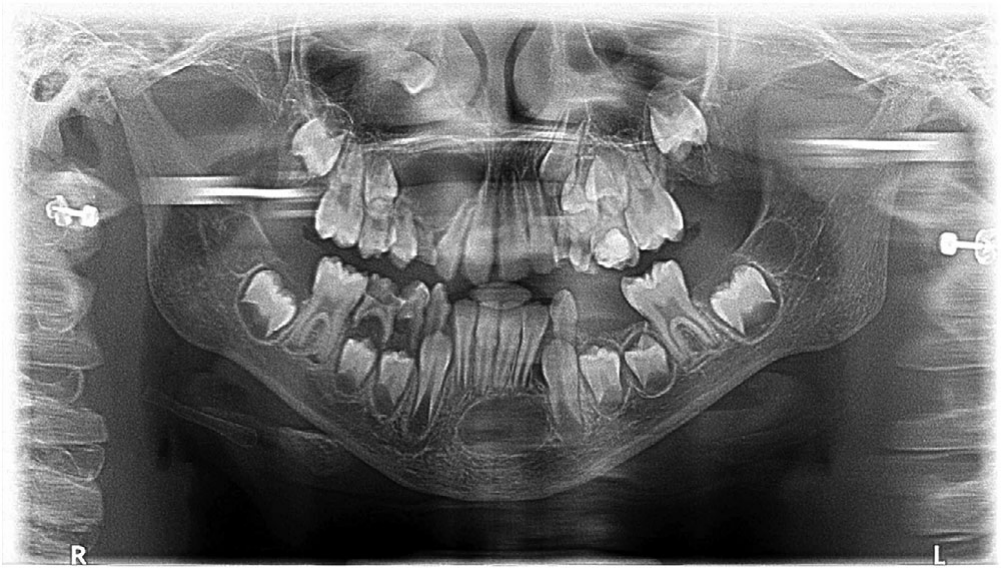
Panoramic radiograph showing the presence of two cysts: one in the mandible and one in the maxilla.

**FIGURE 2:**
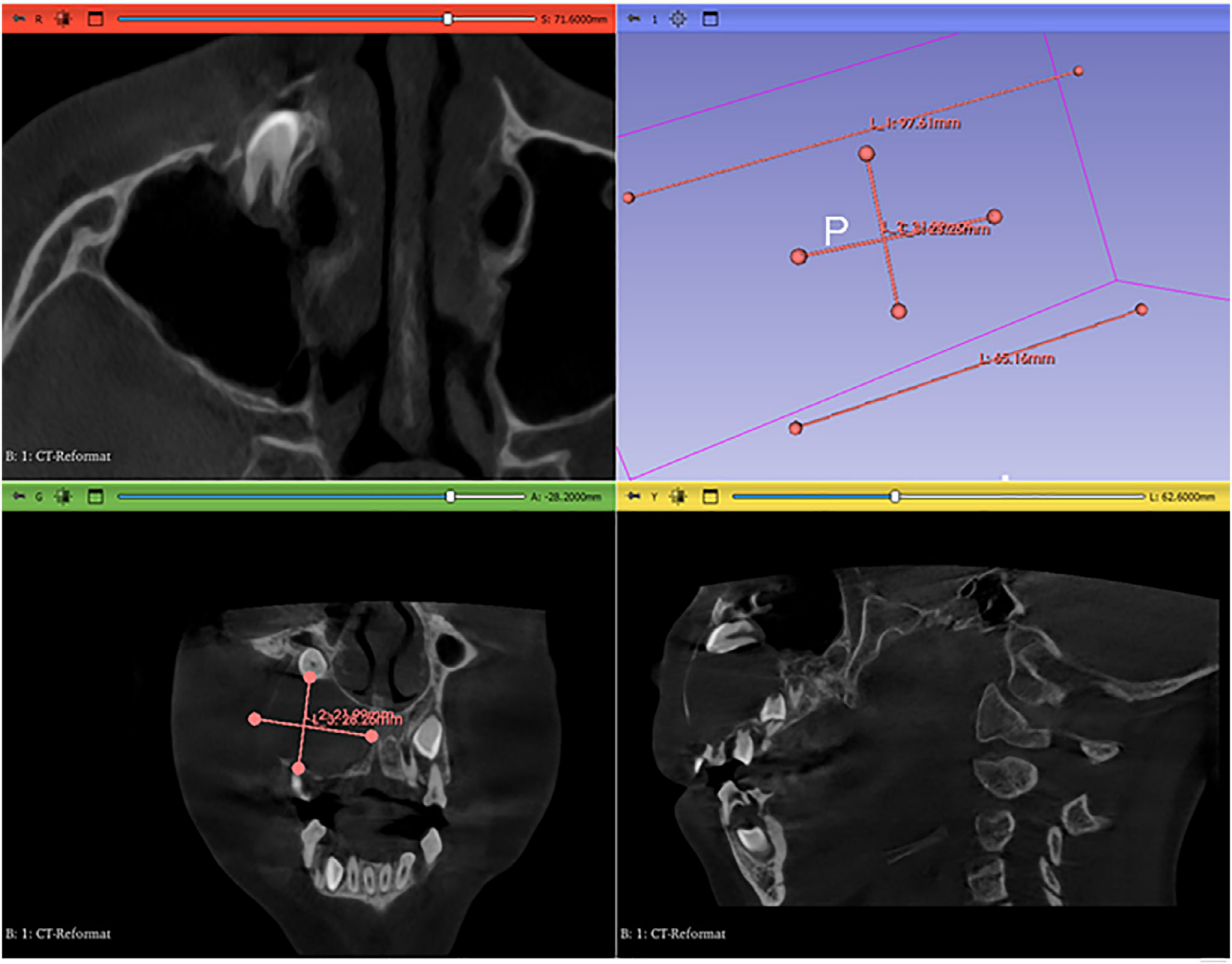
CBCT reconstruction showing the maxillary cyst.

A panoramic radiograph (Figure 1) revealed two radiolucent lesions: one well‐defined unilocular radiolucency in the anterior mandible crossing the midline, and a large radiolucency in the right maxilla around the crown of the impacted canine. To better define the extent of these lesions, a cone‐beam CT (CBCT) scan was obtained. The CBCT (Figure 2) confirmed a 2 cm cystic lesion expanding the mandibular symphysis, extending to the apices of the lower central incisors. The second lesion was a 3.0 cm radiolucent cavity in the right maxilla, centered above the deciduous canine and second premolar area, causing displacement of the permanent canine. The mandibular imaging features were consistent with OKCs (given the Gorlin syndrome context). The maxillary lesion presented several atypical radiological features for an odontogenic keratocyst. While OKCs classically tend to expand in the anteroposterior direction with limited cortical expansion, this lesion showed buccal expansion extending toward the orbital floor and piriform rim. Close relationship with the crown of the displaced impacted canine strongly mimicked a dentigerous cyst, representing a potential diagnostic pitfall. The presence of multiple jaw cysts in a child with Gorlin syndrome supported the diagnosis of syndromic OKCs

### 2.3 Second Anamnesis

After the appearance of these two cysts, Gorlin syndrome was mentioned to the patient's mother. She then told us that she herself had Gorlin syndrome and that her daughter had also been diagnosed with it during early childhood, showing cutaneous manifestations, although follow‐up had been discontinued.

The patient has a limited understanding of french, which makes it difficult for her to explain the nature of the cysts. We wanted to refer her to the maxillofacial surgery department at the university hospital, but she was hesitant. We believed this was mainly because of her lack of understanding.

An explanation using radiographs was attempted, but the mother did not understand the seriousness of the lesions. We therefore decided to take a different approach by showing a 3D‐printed model.

## 3 Material and Methods

### 3.1 3D Model Fabrication

**FIGURE 3:**
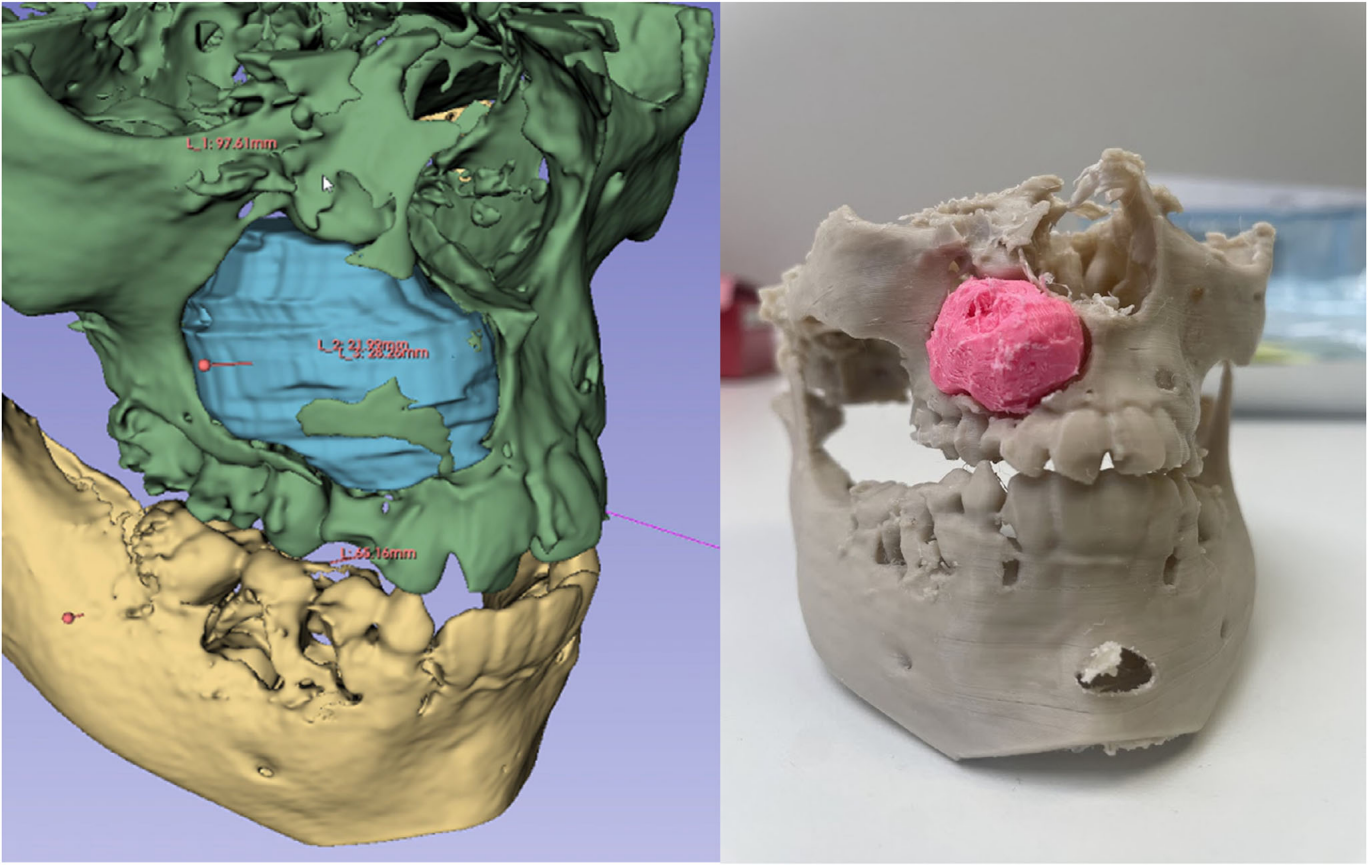
Segmentation of anatomical structures based on CBCT data and corresponding 3D‐printed model showing the mandible and maxillary cyst, with the cyst printed in soft pink PETG.

**FIGURE 4:**
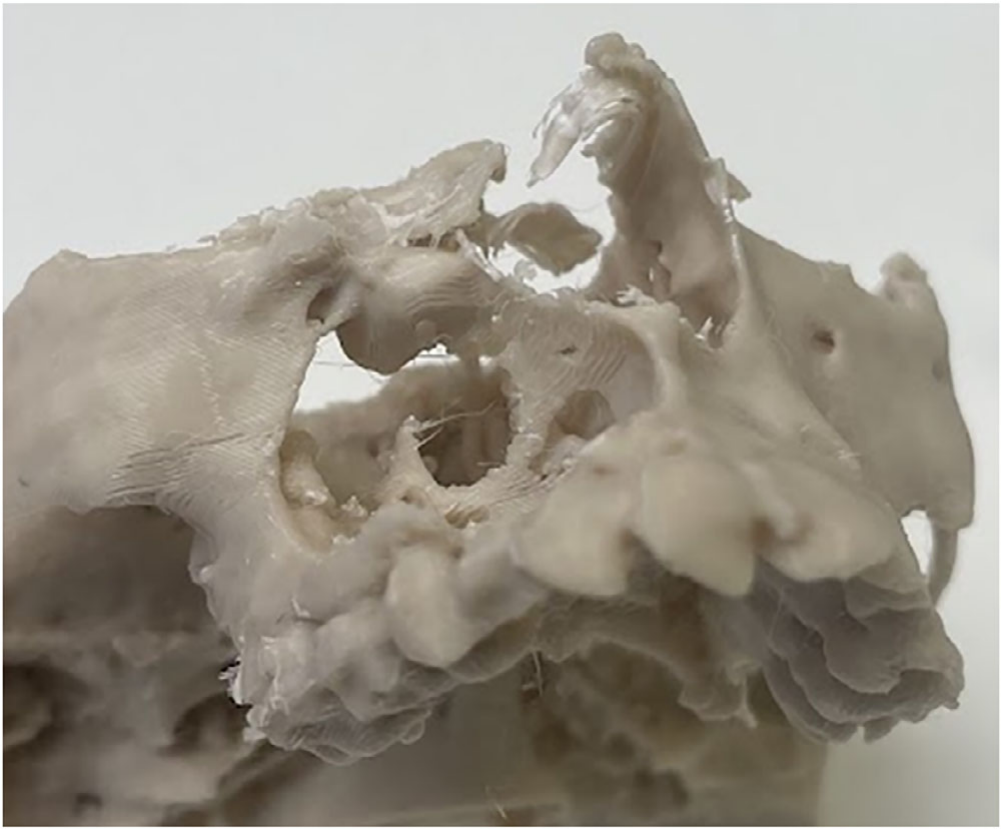
3D‐printed models illustrating the cystic cavity, allowing visualization of the canine located beneath the orbital floor.

Using the DICOM data from the CBCT, a 3D reconstruction of the craniofacial skeleton was created. The digital workflow involved importing the scan into 3D Slicer (an open‐source medical imaging software platform) and segmenting the regions of interest (Figure 3). Threshold‐based and manual segmentation techniques were employed to isolate the jawbones and cystic cavities. Both cysts were segmented as voids within the mandible and maxilla, respectively. The task took three hours to complete by a dentist experienced in medical imaging and 3D software. The segmented structures were exported as STL files. The mandibular model included the cyst in the symphysis, and the maxillary model included the right canine region cyst along with several adjacent teeth and part of the maxilla to show its proximity to the orbit. These STL models were processed for printability (with minor mesh edits were made using Meshmixer) and then fabricated with a desktop fused deposition modeling (FDM) 3D printer (using PLA filament at 0.2 mm layer height and bone color). Two separate models were printed: one of the mandible and one of the maxilla, each approximately life‐size. Cysts were printed in PETG at 0,2 mm in pink color, PETG filament is soft. (Figure 4).

Printing time was about 14 h in total, and the material cost attributed to this specific printing was minimal (just a few euros’ worth of filament).

The models were ready one week after the patient's imaging, underscoring the quick turnaround possible with 3D printing.

### 3.2 Use of 3D Model in Consultation

During the second consultation, we used the 3D‐printed models to explain the patient's cyst. The family visualized how the cyst had expanded the bone between the lower incisors. The maxilla model showed the canine tooth displaced high under the orbit with the cystic lesion surrounding it. Mother and patient were encouraged to handle the models. We pointed out normal anatomical landmarks and then the extent of the cysts, illustrating why an approach through the gums would be necessary to access those areas. By physically demonstrating the cysts’ size and proximity to critical areas (e.g., the developing teeth, the nasal floor, and the orbital floor), the dentist conveyed the rationale for intervention and the potential risks (such as tooth loss or, in the maxilla, the need to preserve the orbital floor's integrity). The family commented that the model made it “much clearer” where and how the surgery would take place, compared to trying to interpret the CBCT slices or 2D drawings. The patient herself, being nine years old, appeared to engage with the model—for instance, she identified her other teeth and understood where the ‘bubbles’ (cysts) were located. This seemed to help alleviate some fear of the unknown; seeing a tangible model of her jaw made the problem more concrete. Informed consent was obtained with the parents to consult at University Hospital, who stated they felt well‐informed about the condition and the possibility of treatment. The model, along with our explanations, helped them understand the importance of regular follow‐up, given the risks of cyst recurrence and dental disease. This greatly facilitated the work of the surgeon we referred them to. He reported that the family had clearly understood the urgency and necessity of intervention and had not missed any of their appointments. No formal feedback was collected from other members of the non‐surgical medical team.

## 4 Discussion

This case illustrates how personalized 3D printing can enhance the care of a pediatric patient in multiple ways. The most notable impact of the printed model was its ability to transform a potentially abstractrive and stressful preoperative discussion into a concrete and accessible exchange. Unlike conventional explanations based on 2D radiographs or on‐screen reconstructions—which are often difficult for non‐specialists to interpret—the physical model allowed the parents to both see and feel the anatomical issue [12]. This tactile aid enhanced their spatial understanding of the cysts’ size and location, and, according to their feedback, made the pathology and its implications much clearer. They could *see and feel* the anatomical problem—a degree of clarity and immediacy that verbal explanations or static images rarely achieve [10]. These benefits are consistent with previous studies that demonstrate 3D‐printed anatomical models enhance patient comprehension and participation in decision‐making, particularly in complex cases. For example, Hadad et al. showed that in maxillofacial trauma scenarios, using a 3D‐printed skull to demonstrate malocclusion improved patient understanding during preoperative counselling [13]. Similarly, Rosenfeld et al. reported that standardized visual tools, including diagrams and physical models, significantly improved parental comprehension during pediatric surgery consent. A recent cluster‐randomized trial in surgical oncology also found that patients counseled with 3D models reported feeling more involved in their care and experienced lower anxiety compared to standard counseling [9]. Patients engaged in this process reported greater satisfaction and experienced less anxiety about their condition and the therapeutic choices available [14, 15].

In our case, although anxiety and comprehension levels were not formally assessed, the family's qualitative feedback aligned with existing findings. The parents appeared more at ease after reviewing the model; their questions became more targeted, suggesting a deeper understanding of the condition and planned intervention, even though the explanation was provided in a non‐native language. They expressed reassurance in being able to concretely visualize pathology.

Another benefit of using the 3D‐printed model was the improvement in trust and rapport. Providing a customized model signaled the thoroughness of our approach and helped convey transparency, reinforcing the family's confidence in the care team [13]. Although genetic follow‐up had previously been discontinued, the family resumed consultations and annual monitoring after our discussion. Parents must entrust their child to surgeons, making this confidence crucial in pediatric care. We believe the model helped build that trust, as evidenced by the parents’ later comments that they “really understood what was going to happen”.

From a practical standpoint, the production of the model was feasible and cost‐effective. The digital segmentation using free software (3D Slicer) and printing on an inexpensive FDM printer meant the only costs were a few hours of labor and minimal material expense. Our in‐house 3D printing workflow made it possible to have the model ready within one week, causing no delay in orientation of the patient. This reflects the growing accessibility of 3D printing, Williams et al. reported that in‐house 3D fabrication of surgical guides and prosthetics in a maxillofacial reconstruction setting eliminated external laboratory wait times and dramatically cut costs. In our case, while the primary goal of printing was patient education rather than creating an implant or guide, the same advantages applied—we could generate the model quickly and cheaply on site. Weng et al. similarly noted that producing 3D models for consent was justified by the dual utility of those models helped both patient's consent and surgical planning [10]. As 3D printing technology continues to advance, we expect that such models will become even more available and customizable. The learning curve for basic medical 3D printing has flattened, and many institutions are now equipped with printers capable of producing anatomically accurate models. In this context, the barrier to implementing 3D models as standard practice for complex cases is lowering.

There are limitations and considerations to note. This is a single case report, and we did not quantitatively assess the impact of the 3D model on the patient's understanding or anxiety. While supported by literature, our impressions of its benefit are still anecdotal. Future studies in pediatric dentistry could formally evaluate parent and patient comprehension with vs. without 3D model aids. Creating a 3D model does require technical resources and time. In our scenario, the digital workflow was handled by a dentist experienced in 3D software, but not all dentists may have immediate access to such expertise. We suggest that the development of 3D printing education in medical and dental curricula will support this. Cost, while minimal for an FDM plastic model, can increase if higher‐fidelity models or larger scales are required. We used two materials for a bicolor model, which was sufficient to convey the relevant anatomy; more complex color or multi‐material prints might enhance realism but also raise costs. Another consideration is infection control and patient safety—the model used in our consent was not a sterile device and was for external demonstration only. It never touch the patient's body, so there were no regulatory concerns in this context (most PLA filaments are non‐toxic and safe to touch).

Despite these considerations, the potential benefits of patient‐specific 3D printed models appear to outweigh their limitations in complex pediatric cases. The model did not alter the diagnosis or surgical management, it improved communication by enhancing understanding, trust, and family engagement. This is particularly valuable in pediatric dentistry, where informed consent relies on parents understanding complex anatomical information. The tangible representation of the lesions made the pathology and treatment rationale easier to understand than conventional imaging alone.

The model also facilitated communication with the child. Rather than causing distress, it encouraged her to explore and understand her own anatomy in an age‐appropriate way. Although these observations remain qualitative, they suggest that patient‐specific 3D models may help reduce anxiety and improve engagement in pediatric patients. Future studies should evaluate these benefits using validated outcome measures.

## 5 Conclusion

This case highlights the potential value of patient‐specific 3D printed models in pediatric dentistry, particularly for complex syndromic conditions such as Gorlin syndrome. The models appeared to improve parents’ and patient's understanding of the lesions, their anatomical extent, and the rationale for surgical management. They also appeared to reduce anxiety and facilitate communication despite language barriers. Family fully adhered to follow‐up recommendations and did not miss any surgical appointments, suggesting improved engagement in care. Beyond surgical planning, low‐cost 3D printed models may therefore represent an effective adjunct for informed consent, therapeutic education, and multidisciplinary communication in pediatric oral healthcare.

## Author Contributions

**Romain Jacq**: Study design, execution, and manuscript writing. **Margaux Picherit**: study design. Anne‐Charlotte Bas: manuscript correction and writing.

## Funding

No funding was received for this work.

## Ethical Statement

The study was conducted in accordance with the principles of the Declaration of Helsinki. Informed consent was obtained from the patient's parents for the use of clinical data and images for publication.

## Consent

Informed consent was obtained from the patient's parents for the use of clinical data and images for publication

## Conflicts of Interest

The authors declare that they have no conflicts of interest related to the content of this manuscript.

## Data Availability

All data generated or analyzed during this study are included in this article. Further inquiries can be directed to the corresponding author.
